# Paraneoplastic Dermatomyositis Associated with Metastatic Seminoma

**DOI:** 10.1155/2016/7050981

**Published:** 2016-05-31

**Authors:** Hidekazu Yoshie, Ryuto Nakazawa, Wataru Usuba, Hiroya Kudo, Yuichi Sato, Hideo Sasaki, Tatsuya Chikaraishi

**Affiliations:** Department of Urology, St. Marianna University School of Medicine, Kawasaki 216-8511, Japan

## Abstract

We report the first case in Japan of paraneoplastic dermatomyositis with pure seminoma, a tumor that extremely rarely accompanies dermatomyositis. The patient presented to the hospital with muscle weakness and erythema and was diagnosed with dermatomyositis from skin biopsy. Routine radiological screening revealed testicular tumor and massive lymph node metastases. We initially performed orchiectomy along with conventional immunotherapy. However, muscle weakness gradually worsened, and he eventually showed dysphagia and forced respiration and became bedridden. Although he seemed close to being too unstable to tolerate further treatment, we started carefully adjusted chemotherapy comprising 4 courses of etoposide plus cisplatin, which proved highly successful. Lymph node metastases completely disappeared and swallowing and respiration fully normalized after completing chemotherapy. We believe that this clinical success was due to our decision to initiate chemotherapy even in such a weak patient.

## 1. Introduction

Dermatomyositis is a connective tissue disorder caused by perivascular inflammation in the skeletal muscles. Clinical manifestations include muscle weakness and skin lesions such as heliotrope rash and Gottron's papules. Approximately 20% of patients with dermatomyositis reportedly show concomitant malignancies, including breast, colon, ovarian, and lung cancers [1].

We recently encountered a dermatomyositis patient with metastatic seminoma, which is extremely rare as an accompanying tumor. Of note was the finding that symptoms of dermatomyositis did not resolve with orchiectomy and steroids but resolved fully with chemotherapy started despite the unstable condition of the patient. We report here the unique clinical course of this patient with dermatomyositis accompanied by metastatic seminoma along with a review of the literature.

## 2. Case Report

A 70-year-old man presented to the Department of Internal Medicine at our hospital complaining of muscle weakness of the extremities, erythema, and impaired gait, which had rapidly deteriorated since 1 month earlier. Muscle weakness was assessed as MMT 2/5, and he was bedridden with PS grade 3. Based on these clinical findings, dermatomyositis was suspected. Skin biopsy confirmed the diagnosis of dermatomyositis.

**Figure 1:**
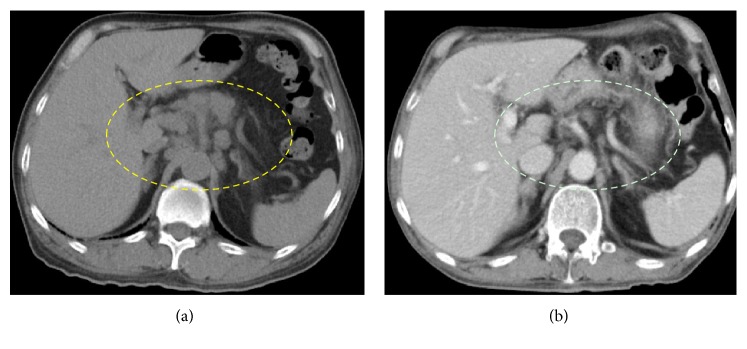
(a) Abdominal CT showing enlarged para-aortic lymph nodes. (b) After four courses of EP, enlarged lymph nodes have completely disappeared.

Whole-body CT was performed as a routine screening for neoplasm, revealing a lesion in the left testis and systemic enlargement of the lymph nodes (Figure 1(a)). The patient was therefore referred to the Department of Urology with a diagnosis of paraneoplastic dermatomyositis with testicular tumor.

Serum levels of CK and aldolase, both representing myogenic enzymes that serve as serum markers for dermatomyositis, were increased to 2621 U/L and 19.1 IU/L, respectively. In terms of markers for testicular tumor, serum LDH was elevated to 1033 U/L, whereas HCG-*β* and AFP levels were within the normal ranges of <0.1 ng/mL and 4.6 ng/mL, respectively.

**Figure 2:**
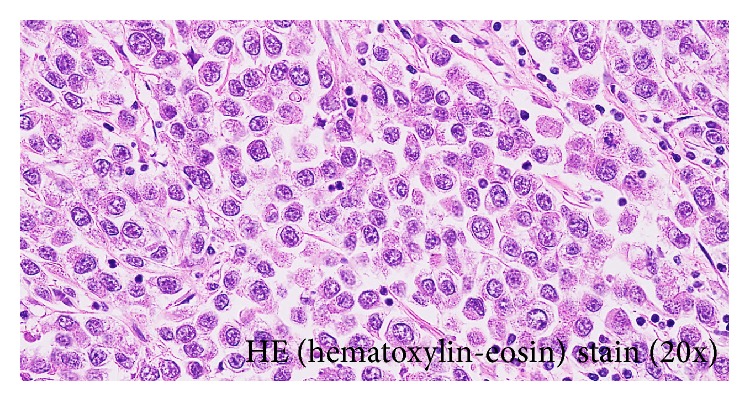
Hematoxylin-eosin staining shows large, oval tumor cells with clear, round nuclei and clear cytoplasm. The tumor was histologically diagnosed as seminoma. Immunological staining showed positive results for placental alkaline phosphatase and C-kit but negative results for AFP and HCG. CD20 and CD3, as markers of lymphoma, were also negative.

On the assumption that the presence of the testicular tumor had played a role in the development of muscle weakness, we performed left orchiectomy. The resected testicular tumor was pathologically diagnosed as seminoma (pT2N2M1aS1: stage IIIB) (Figure 2). However, 10 days after orchiectomy, muscle weakness had not improved and instead showed gradual worsening. PS deteriorated to grade 4. At this point, the patient showed symptoms of dysphagia. As he seemed too weak to receive anticancer chemotherapy, we selected administration of steroid pulse therapy and IVIg, as a standard immunotherapy for dermatomyositis. During treatment, the muscle weakness progressed further, and he eventually showed forced respiration.

**Figure 3:**
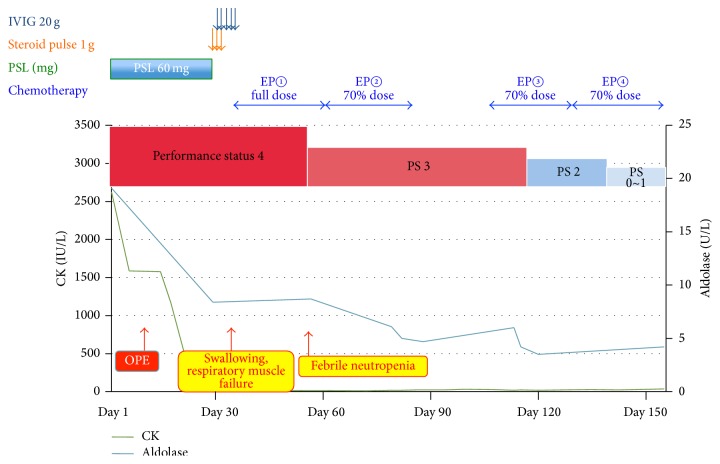
Clinical course of the patient. After orchiectomy and conventional immunotherapy for dermatomyositis, PS remained low (PS grade 4) and levels of myogenic enzymes were not fully normalized. We then started four courses of EP regimen, and at the time of completion, PS had improved to grade 0 or 1, and MMT became 5/5.

Since removal of the tumor along with immunotherapy had not relieved his symptoms, we were left with no choice but to treat the patient with chemotherapy for seminoma. This had not been the initial option, as the patient was considered almost too weak to tolerate this therapy. We selected four courses of EP regimen, comprising daily doses of etoposide at 100 mg/m^2^ and cisplatin at 20 mg/m^2^ for 5 consecutive days every 3 weeks, excluding bleomycin from the standard regimen, because dermatomyositis could have been complicated with interstitial pneumonia. The treatment course is shown in Figure 3.

In the first course of the EP regimen, abdominal lymph nodes showed a mild reduction in size and respiratory muscles seemed to regain some strength. Because of bone marrow suppression with this first course, the doses of both cisplatin and etoposide were reduced to 70% from the second course onward.

By the time all four courses of EP had been completed, the metastatic lymph nodes had completely disappeared (Figure 1(b)). In addition, PS had improved to grade 0 or 1, and MMT had reached 5/5. Swallowing and respiration seemed fully normal, and he was discharged from the hospital, walking independently.

## 3. Discussion

Breast, colon, lung, and ovarian cancers are frequently reported as associated cancers in paraneoplastic dermatomyositis, but association with testicular tumor has rarely been documented [1]. Our review of the literature identified 13 cases with testicular tumor [2–4]. Most were associated with nonseminoma, whereas only 3 cases were accompanied by pure seminoma [4–6]. Our case with pure seminoma represents the first such case in Japan and also the oldest patient with this pathology in the literature.

Dermatomyositis has been reported in various stages from stage I to stage III [2–4]. In our case, testicular tumor was very advanced, with massive lymph node metastases.

Autoimmune reactions have been speculated to play a role in the mechanisms of paraneoplastic DM. Kaji et al. recently identified an autoantibody reactive with 155/140 kDa nuclear proteins, which could serve as a serological marker for the disease [7]. They reported that levels of this autoantibody are increased with increasing clinical severity of the disease and decrease after successful treatment [8]. They speculated that presence of the tumor promotes production of the autoantibody, in turn causing progression of myositic symptoms. Reducing levels of the autoantibody by removing the tumor, in the form of not only the primary lesion but also any metastatic lesions, thus seems reasonable, and we consider that our favorable results can be explained based on this assumption.

In the present case, symptoms of dermatomyositis progressed despite removal of the primary tumor along with immunotherapy. Although our patient appeared too weak to tolerate chemotherapy, which is not generally performed for such fragile patients with PS grade 4, we had no other choice but to apply this option to combat the metastatic lesions.

Our regimen, which was carefully selected and dose-adjusted, proved successful. At the completion of chemotherapy, symptoms of dermatomyositis had fully resolved and lymph nodes had totally disappeared, indicating that our strategy was valid. Although administration of anticancer agents to highly unstable patients with poor PS is a challenge, we consider that such treatment could be feasible under an appropriate regimen with adjusted doses. The patient in this case is now being followed up on an outpatient basis. As of 2 years since being discharged from hospital, the patient has experienced no recurrence of the testicular tumor or DM.

## Abbreviations

MMT: Manual muscle testing
PS: Performance status
CT: Computed tomography
CK: Creatine kinase
LDH: Lactate dehydrogenase
HCG-*β*: Human chorionic gonadotropin-beta
AFP: Alpha-fetoprotein
IVIg: Intravenous immunoglobulin
EP regimen: Regimen of etoposide plus cisplatin.
